# Multisectoral Lessons from Healthy Communities

**Published:** 2010-10-15

**Authors:** Mary A. Pittman

**Affiliations:** Public Health Institute

## Abstract

The healthy communities movement can provide insight into population health efforts in the United States, particularly in the context of recent health care reform. The movement has evolved from multisector partnerships that focused on improving the health, well-being, and quality of life for people and the social determinants of health to partnerships that focus more on chronic disease prevention, health equity, and environmental change. Evaluating the effects of community programs on population health has been challenging for a number of reasons. More metrics need to be developed for population health that will address inequities and focus policies on long-term health effects.

## Healthy Communities as a Population Health Strategy and Social Change Model

The healthy cities and communities movement provides a context for developing and reviewing population health efforts. The healthy cities movement in Europe predated and informed the healthy cities and communities movement in the United States; the concept grew from a premise that "cities must be looked at as interrelated complex ecological organisms in which housing, transport, city planning, economic development, and many other facets interacted with health and medical issues" (1). The World Health Organization adopted Healthy Cities in 1987 (2) when 11 healthy city pilot projects were launched, and approximately 1,200 cities and towns from 30 countries were participating by 2008, moving from individual projects to a movement with coordinated efforts with common goals.

In the United States, healthy communities partnerships were convened by public and private health care and public health organizations, municipalities, foundations, and local civic organizations. They typically sought to build local support for health improvement activities by engaging diverse partners around a shared vision and a collaborative agenda that included multisectoral systems change. Bethel New Life in Chicago is an example of business and faith communities coming together in a grassroots effort that addressed the environment and later included jobs as well as improvements in housing and health (3). Equally effective were top-down efforts driven initially by funders, or elected officials and sideways-initiated efforts when community-based organizations initiated the efforts with government or businesses. Local context, community assets, and priorities drove the work of these partnerships, but, for sustainability and transformation from an initiative to a local movement, there had to be shared power. In many cases, partnership objectives included not only specific improvements in health but also development of community resources, capacities, and policies oriented to improve health. In this article, I will discuss how the healthy communities movement influenced current population health policies in the United States.

## Multisectoral Partnerships: the 1990s to the Present

In the 1990s, multisectoral partnerships became more influential; such partnerships were voluntary agreements between 2 or more people or entities to work collaboratively toward a shared outcome. Prominent examples were 1) the Community Care Network Demonstration among hospitals, health care organizations, and community group representatives from business, education, and religious organizations, and 2) the Turning Point Initiative, a partnership of the public health sector and community organizations. These programs were fueled by investments from private foundations and government agencies as a result of changes in state and local responsibility for health care programs. Also aiding this growth was increased recognition of the contributions of systems thinking (a way of understanding the relationships among a system's parts) and the social determinants of health (the importance of social factors such as income and where one lives in determining an individual's health) (4).

Multisectoral partnerships have exhibited some consistent patterns and themes, including strong distributed leadership in which no single individual or organization is the appointed leader on all issues but everyone shares in the governance. Often a charismatic leader may initiate the effort, but sustainable initiatives require broader leadership and transparent governance and decision-making processes with identified and, ideally, funded staffing. The very structure and leadership of a collaboration can determine the types of initiatives that are undertaken.

The initiatives typically have a health status improvement focus, informed by the social determinants of health. Classically, the initiatives take the form of multisectoral public-private collaborative partnerships focused on measurably improving the health and well-being of people, the quality of life, and the social determinants of health in the communities in which they live. Unlike organizational programs that address symptoms, these partnerships provide local communities with proven strategies and models to create and sustain positive, lasting policy changes for healthy living.

Such endeavors have been complemented by growing governmental efforts to help bring about reform by creating indicators and setting public goals to enhance health and avoid disease. Many states adopted or developed state-level *Healthy People* (5) goals; awareness and use of the goals extended beyond public health agencies into health care providers and community organizations. One lesson from community initiatives is that metrics — measures of performance — help guide local efforts to address problems defined by the community and provide accountability and transparency to the work being done. Metrics, such as the number of children on school lunch programs and walkable routes to school, have been connected to interventions addressing childhood obesity in a community (6,7). Metrics also help create a constituency for local political support and policy change. In 2002, indicators based on multiple metrics about parents reading to their children influenced a coalition to support and promote reading among clients in the federal Special Supplemental Nutrition Program for Women, Infants, and Children (WIC) program in Seattle-King County, Washington (8).

## Current Initiatives and Trends

Although most of the population health initiatives of the 1990s have concluded, approaches in the 2000s focus more on chronic disease prevention, health equity, and environmental change strategies. CDC's ACHIEVE communities (Action Communities for Health, Innovation, and EnVironmental ChangE), which by 2013 will have 200 participating communities, are leading examples for new prevention models for health care reform (9). Communities Putting Prevention to Work, which received $650 million through the federal American Recovery and Reinvestment Act to focus on obesity and tobacco use, builds on programs such as ACHIEVE to produce measureable outcomes from community collaboration. Kaiser Permanente, the nation's largest integrated delivery system and a leader in the healthy communities movement, identified 10 design principles for multisectoral community work. The principles are based on the emerging evidence base and Kaiser Permanente's experience working with community partners. These principles are consistent with those of other preeminent healthy communities and are part of Kaiser's community benefit work (10).

## Evidence for Action

Determining the effectiveness of community programs can be difficult because of changes in leadership, participants, resource allocations, and external environmental factors as well as the dynamic nature of the communities in which these programs are embedded. Limited data systems, resources, or technical expertise to implement comprehensive evaluations also hinder measurement of effectiveness. With these challenges, evaluators have not been able to link healthy community or multisectoral community-based partnerships to overall improvements in population health, in part because few evaluation time frames are long enough to capture distal measures of health outcomes. Health information technology resources being developed to implement health reform can also inform community programs. That said, lessons from community-based initiatives show proximal and intermediate process measures (ie, a reduction in emergency department visits for ambulatory care sensitive conditions such as asthma or pneumonia, or an increase in screening rates) that can inform future health systems work.

Conrad and colleagues (11) described 3 lessons in their evaluation of the national Community Care Network (CCN) Demonstration. The project's 25 public-private partnerships in communities around the nation were responsible for addressing access to health care and lack of health insurance and for focusing on community prevention and the health of residents with the fewest resources. The American Hospital Association's Health Research and Educational Trust (12) managed and disseminated the findings from the project funded by the W.K. Kellogg Foundation. The evaluation concluded that although the sites did not measurably reduce health and social service costs in their communities, they achieved some of their objectives, particularly in the areas of community health focus and community accountability. However, few of the partnerships crafted the kind of population-based information systems needed to track community health outcomes or the tradeoffs in reallocating resources among competing uses in the community as a whole. New information tools will facilitate these processes in the future.

The lessons from the CCN Demonstration and some examples of health improvement initiatives can be summarized as follows:

- **Lesson 1: Community-based initiatives are less likely to produce measurable results in health behavior unless the program *unpacks* the broad-focused community intervention into its various parts and continually measures progress on those component parts and their contribution to the larger goal of community health improvement.** This finding by Conrad and colleagues (11) is consistent with the message that smaller visible wins are necessary to keep a collaborative process engaged and working toward larger goals. Broad, vague goals without measures to show progress along the way are challenging to sustain. According to Wagner and colleagues (13), the Kaiser Family Foundation Community Health Promotion Grant Program in the western United States and the CCN Demonstration faced similar challenges in many of their demonstration sites.
- **Lesson 2: Focused interventions are more likely to produce community health improvement if they are targeted to a clearly defined community population and implemented and managed by a small number of accountable organizational entities.** The Community Health Promotion Grant Program evaluation by Wickizer et al (14) emphasized the importance of clear processes and theories of interventions and accountability to the community. Examining this same initiative, Wagner (13) found a general failure to achieve the targeted health outcomes and suggested that future "efforts should focus on developing theories and methods that can improve the design and evaluation of community-based interventions." The Healthy Carolinians initiative of the Turning Point program that supported both state and local policy change around healthy communities identified 4 success factors in their community health initiatives: gaining communitywide buy-in, establishing and maintaining data-driven decisionmaking, involving the community to ensure community-determined priorities, and collaborative interventions and evaluations (15). In their comprehensive review of more than 2 decades of collaborative partnerships, Roussos and Fawcett (16) found some notable population-level outcomes for conditions amenable to short-term impact. For example, although not strong enough in the authors' view to draw conclusions about the effects of partnerships on population level outcomes, a partnership that focused on 1 objective with short-term impact resulted in a 43% reduction in lead poisoning in New York City within 4 years, following 10 years of higher rates before the partnership.
- **Lesson 3: The broader the intervention focus and the more varied the target population, the more separate program components will need to be integrated to achieve positive community health outcomes.** The Turning Point program evaluation by Baxter (17) stressed building and integrating capacity within partnerships by creating strategic links and engaging in collaborative decision-making processes driven by scientific evidence. Cheadle et al (18) evaluated the California Wellness Foundation’s Health Improvement Initiative in communities with broad-based partnerships. Volunteerism alone was found to be insufficient to create community-level systems change; rather, a well-supported infrastructure was critical to success. Lasker and Weiss (19) concluded that the potential value of a diverse group of people in a community health collaborative is enhanced by the following: 1) obtaining more accurate information about community concerns and priorities; 2) helping participants understand how different programs and services do or can interrelate; 3) combining statistical and qualitative information to understand the root causes of problems and create potential solutions; and 4) providing a broader understanding of the local history, culture, values, and politics. In a follow-up study of community participation in 5 partnerships, Lasker and Guidry (20) found that people most affected by a problem, who could give the most insight into it, are usually marginalized by the process and have little voice in determining what will be done to help them. To achieve the “promise of community participation,” processes need to be created to include these historically excluded people, giving them “influence where it counts.” Community participation research has focused on methods that include as much of the community as possible (21).

## Incentives for Change

The community or population health approach is gaining interest in many policy sectors because the lack of health care coverage for millions of people and the cost of health care have raised fundamental concerns:

- Are our public and private investments and policies aimed at optimizing population health outcomes and eliminating disparities?
- With health reform upon us, there are additional questions about whether the monetary and other incentives in the health care system, and other systems that directly affect and provide cobenefits to health status (such as agriculture, education, jobs, and energy), are aligned with producing improved health outcomes.
- Have we unwittingly ignored and externalized the causes of ill health, allocating most of the financial rewards in the health system solely to treating disease?

As a nation we finally have health reform that moves beyond the finance and delivery of care services and can embrace the science and practice of prevention and the determinants of health. As long as incentives and reimbursements in the health care system remain primarily tied to treating diseases rather than promoting health outcomes, we will never effectively address (or properly encourage and reward) what contributes to good health in the first place.

## Investing in Health

Given the rising costs of chronic disease, it is instructive to examine the drivers or underlying forces behind the leading causes of death — smoking, poor diet, physical inactivity, and other contributors such as lifestyle, behaviors, and socioeconomic status (22). The health field model (23) provides a framework for examining the effects these factors have on health and premature deaths. Poverty and lack of education are among the most substantial drivers of poor health and premature death (24). Consequently, the greatest leverage point to addressing health outcomes is a focus on social policy and environmental factors.

If we agree that population health is a societal investment, guidelines and metrics should be developed with a national agenda for investment that takes into account the variation in the levels at which communities start the improvement process. This places America's communities — and their role in advancing public policies that affect the determinants of health — at the heart of the solution and the locus of positive change. Improvements in population health are inextricably linked to the health of the community environments where we live, love, work, shop, eat, go to school, and worship. The factors that build people's health are the same factors that build the health, wealth, safety, and vitality of families and communities.

A more integral world view and new approaches for measuring return on investment to local, state, and national priorities are essential to identify direct and demonstrable cost savings and revenue contributions associated with improvements in population health. When rooted in local-level entrepreneurship, new investments in businesses that have social dividends concurrently stimulate the economy, reduce poverty and violence, and save billions of dollars in costs to the health care and criminal justice systems. These are the kinds of investments that produce the quality of human capital needed to stimulate and drive our postindustrial economy.

## Conclusion

Community coalitions voice a common refrain: "How do we connect what we are envisioning and prioritizing locally with state and national policy-making processes?" In effect, they are naming the frustrating chasm between local and regional civic governance processes and policy processes in statehouses and in Washington, DC. This is a chasm that President Barack Obama vowed to bridge in general and specifically in the American Recovery and Reinvestment Act and the community prevention funding committed to health reform.

Whether at the level of personal decision, corporate practice, or collaborative partnership, building a healthier community has become an expressed priority across the country (25). Lessons from past population health improvement efforts suggest that to achieve demonstrable health improvements, community initiatives will need to have the following:

- a clearly defined vision for well-understood problem(s) for which there are measurable goals, evidence-based intervention strategies, and shared accountability for success;
- a disciplined focus on a small number of goals;
- a socioecologic approach that affects multiple aspects of the issue through multiple stakeholders;
- support for the infrastructure, including data, to implement successfully; and
- an intervention that lasts long enough to create a sustainable change.

Chronic illness prevention and inequities in health status are 2 fruitful starting points for population health efforts. Other leverage points with momentum and enthusiasm include implementation of health care reform; new interests of specific sectors (eg, hospital community benefit and businesses' focus on costs and productivity); social networks; and environmental health awareness.

Learning from case studies and limited evaluations offers insight into actions that can sustainably improve economic, ecologic, social, and population health at all levels and can be integrated into efforts to reform health care in the United States. However, more research-based evidence is needed concerning how to spread effective population health interventions and how to evaluate their return on investment. We have never been in a better position to integrate financial incentives for population health than we are today. The Obama administration's commitment to changing the status quo of inequitable health in the United States and multisectoral leadership can improve the health of all Americans. Now is the time to develop and test incentives and mechanisms that will prioritize population health outcomes.
